# Adapting ethical guidelines for adolescent health research to street-connected children and youth in low- and middle-income countries: a case study from western Kenya

**DOI:** 10.1186/s12910-015-0084-y

**Published:** 2015-12-18

**Authors:** L. Embleton, M. A. Ott, J. Wachira, V. Naanyu, A. Kamanda, D. Makori, D. Ayuku, P. Braitstein

**Affiliations:** College of Health Sciences, School of Medicine, Moi University, Eldoret, Kenya; Dalla Lana School of Public Health, University of Toronto, Toronto, ON Canada; Department of Pediatrics, Indiana University School of Medicine, Indianapolis, IN USA; Academic Model Providing Access to Healthcare (AMPATH), Eldoret, Kenya; College of Health Sciences, Department of Behavioral Sciences, Moi University, Eldoret, Kenya; Moi Teaching and Referral Hospital, Eldoret, Kenya; Fairbanks School of Public Health, Department of Epidemiology, Indiana University, Indianapolis, IN USA; Regenstrief Institute, Inc., Indianapolis, IN USA; Division of Epidemiology, 155 College Street, Toronto, M5T 3M7 ON Canada

**Keywords:** Street children and youth, Ethics, Research, Consent

## Abstract

**Background:**

Street-connected children and youth (SCCY) in low- and middle-income countries (LMIC) have multiple vulnerabilities in relation to participation in research. These require additional considerations that are responsive to their needs and the social, cultural, and economic context, while upholding core ethical principles of respect for persons, beneficence, and justice. The objective of this paper is to describe processes and outcomes of adapting ethical guidelines for SCCY’s specific vulnerabilities in LMIC.

**Methods:**

As part of three interrelated research projects in western Kenya, we created procedures to address SCCY’s vulnerabilities related to research participation within the local context. These consisted of identifying ethical considerations and solutions in relation to community engagement, equitable recruitment, informed consent, vulnerability to coercion, and responsibility to report.

**Results:**

Substantial community engagement provided input on SCCY’s participation in research, recruitment, and consent processes. We designed an assent process to support SCCY to make an informed decision regarding their participation in the research that respected their autonomy and their right to dissent, while safeguarding them in situations where their capacity to make an informed decision was diminished. To address issues related to coercion and access to care, we worked to reduce the unequal power dynamic through street outreach, and provided access to care regardless of research participation.

**Conclusions:**

Although a vulnerable population, the specific vulnerabilities of SCCY can to some extent be managed using innovative procedures. Engaging SCCY in ethical research is a matter of justice and will assist in reducing inequities and advancing their health and human dignity.

## Background

Street-connected children and youth (SCCY) live with a variety of health-related risks [1]. Reviews demonstrate gaps in knowledge about SCCY’s health [1, 2] and limited evidence on risk reduction or health promotion interventions for this population, particularly in Iow- and middle-income countries (LMIC) [3]. These gaps are due, in part, to the ethical challenges in engaging this vulnerable population in research.

While all children have research-related vulnerabilities [4], SCCY in LMIC are a particularly marginalized population that have unique and often intersecting vulnerabilities including absence of a parent/guardian to provide consent, uncertain cognitive capacity, high rates of illiteracy and substance use, vulnerability to coercion, lack of basic necessities, access to health care, and significant human rights violations. Ethical conduct of research activities within LMIC are beset by an additional set of social, cultural, and economic issues affecting informed consent and assent, community participation, standards of care, and equity [5–7]. Together, these pose significant challenges related to SCCY’s involvement in research and result in an understudied and underserved population [8, 9]. However, research is essential to advance the health and human dignity of SCCY, and to develop solutions to issues relevant to their complex physical and psychosocial health and lives is of paramount importance. The core universal ethical principals of beneficence, justice and respect for persons [10] provide an important framework to guide the conduct of ethical research with this vulnerable population in low- and middle-income settings.

While international research ethics guidelines provide increased protections for children, they also discourage exclusion of vulnerable groups as a matter of access and justice. The Declaration of Helsinki states that, “Groups that are underrepresented in medical research should be provided appropriate access to participation in research,” [11] and The International Ethical Guidelines for Biomedical Research Involving Human Subjects similarly states that, “groups or communities to be invited to be subjects of research should be selected in such a way that the burdens and benefits of the research will be equitably distributed. The exclusion of groups or communities that might benefit from study participation must be justified” [10]. Exclusion from research activities denies SCCY their right to participation in issues affecting their lives as provided under the UN Convention of the Rights of the Child [12], and undermines the core ethical principles of justice [10].

The international Ethical Research Involving Children (ERIC) project provides a comprehensive framework for undertaking research with children and youth [13], and the Society for Adolescent Health has produced *Guidelines for Adolescent Health Research* [14]. However, there are no specific guidelines for the ethical conduct of research with children and youth in street situations, although there are millions of them [15]. In both high- and low-income settings, researchers have recognized specific challenges to SCCY participating in research [9, 15–19]. Due to their complex circumstances and vulnerabilities, participation in research by SCCY in LMIC requires additional considerations that are responsive to their needs and the social, cultural, and economic context, while upholding core ethical principles of respect for persons, beneficence, and justice [10].

As part of three interrelated research projects in western Kenya with SCCY [20–25], we created procedures to address the multiple vulnerabilities related to research participation within the local context. The objective of this paper is to describe these processes of adapting ethical guidelines for SCCY’s specific vulnerabilities in LMIC.

## Methods

### Setting

Eldoret is Kenya’s 5th largest town and the administrative centre of Uasin Gishu (UG) County. It is home to Moi University (MU), Moi Teaching and Referral Hospital (MTRH), and the Academic Model Providing Access to Healthcare (AMPATH) program, one of the world’s largest HIV care providers [26]. Approximately 51.3 % of the population in UG County live below the poverty line and 52 % of the population are below the age of 20 [27]. Post-election violence, rapid urbanization, abject poverty, and HIV/AIDS have contributed to the existence of children on the streets of Eldoret [28–30].

#### Research projects

We included 446 SCCY across the three studies based in Eldoret. Five were found to be ineligible at screening, and one failed the comprehension test.

*Study 1. Street Children & Substance Abuse: Knowledge, Attitudes and Practices (KAP) in Kenya*: The primary objective of this study was to describe SCCY’s substance use KAP. Using both venue-based and snowball sampling, 146 SCCY aged 10–19 years participated in the cross-sectional survey, 40 were invited to participate in focus group discussions (FGD), and 30 returned voluntarily for the FGD. This study received ethical approval from the Indiana University (IU) Institutional Review Board (IRB), the University of Toronto Research Ethics Board, and the MU and MTRH Institutional Research Ethics Committee (IREC). All ethics committees approved the consent procedures. Approval for the study was also provided by the District Children’s Officer (DCO), and we obtained a waiver of individual guardian consent because as per human subjects regulations, the study was minimal risk, the study could not have been practicably carried out without the waiver, and because the waiver did not adversely alter the risk-benefit ratio for participants. All prospective participants underwent a comprehension assessment to ensure the SCCY understood the nature of their participation in a research study, the study procedures, and their right to withdraw at any time or withhold any answers. Individual written assent was obtained from each participant. Assent was obtained by a social worker trained in assenting vulnerable populations (especially children). Children requesting or requiring healthcare but who were not eligible to participate in the study or who refused to provide assent were provided with healthcare services without enrolment into the study. Additional details on study methods can be found in the respective publications [20, 21].

*Study 2. The Sexual and Reproductive Health Study:* The objectives of this study were to characterize the sexual health of SCCY, estimate the prevalence of STI’s including HIV, and identify factors associated with prevalent infections. Participants were recruited and enrolled by street outreach workers until the target sample size of 200 participants was met. Study procedures included a questionnaire and screening for STI’s and HIV. This study received ethical approval from MU and MTRH IREC, the IU IRB, and the Miriam Hospital Institutional Review Board. All ethics committees approved the consent procedures. Approval for the study was also obtained from the DCO in Eldoret. All prospective participants underwent a comprehension assessment to ensure the SCCY understood the nature of their participation in a research study, the study procedures, and their right to withdraw at any time or withhold any answers. Those prospective participants passing this screening phase were enrolled; those over 18 provided informed consent. Those under 18 provided assent in the presence of a child advocate. There were two prospective participants who failed to meet the criteria for understanding and were not enrolled. None refused participation in the study. Additional details on study methods can be found in the respective publications [23, 31].

*Study 3. The Orphaned and Separated Children’s Assessments Related to their Health and Well-Being Project:* The objective of this study is to evaluate the effect of care environment on children’s physical and mental health [24, 25]. The cohort includes 300 households, 19 Charitable Children’s Institutions, and 100 SCCY in UG County. The study began enrolling participants in June 2010. Research procedures include baseline and semi-annual physical exams, interviews, and surveys of mental health, physical health, and social determinants of health. The MU and MTRH IREC and the IU IRB approved this study. All ethics committees approved the consent procedures. Approval for the study was also obtained from the District Children’s Officer (DCO) in Eldoret. All prospective participants underwent a comprehension assessment to ensure the SCCY understood the nature of their participation in a research study, the study procedures, and their right to withdraw at any time or withhold any answers. Informed consent was provided by the DCO for SCCY. Individual written assent was provided by SCCY. Fingerprints were used for SCCY unable to sign or write their name. Additional details on the study methods can be found in the respective publication [22].

#### Identifying ethical considerations

The Society of Adolescent Health Guidelines for Adolescent Health Research [14] and the international ERIC project [13] provide comprehensive guidelines for conducting ethical research with children and youth. These guidelines provide a universal framework for the basis of ethical considerations in relation to adolescent health research and are applicable to research being conducted in LMIC to safeguard participants. We utilized these guidelines to identify and guide ethical considerations in relation to research with SCCY in Kenya. We also worked with our local and international institutional research ethics boards, ethicists, and the community to identify areas that required special considerations, such as informed consent and assent, and to develop ethical approaches that safeguarded participants within the local sociocultural context. Three key categories emerged through these consultations and include: 1) community engagement and equitable participation, 2) informed consent and assent, and 3) vulnerability to coercion and responsibility to protect. The overarching principles of ethical research, justice, respect for persons, and beneficence, provided a framework to identify the main challenges and vulnerabilities of street-connected children and youth’s participation in research. We situated the associated ethical considerations and approaches our team identified, adapted and utilized within this framework, as demonstrated in in Table 1.

**Table 1:** The challenges and vulnerabilities of SCCY participating in research activities, the associated ethical considerations and approaches to mitigate risks

Guiding principle	Challenges & vulnerabilities	Ethical considerations	Approaches & Recommendations
Justice	Socio-cultural context	- What is appropriate within the local context in relation to research processes?	- Community engagement and participation
		- What degree of autonomy do children have locally?	- Embedding research into existing programs and systems to build capacity.
		- What are the community’s views on issues of street children?	
	Equity	- Participation in the research process, input into activities and research development	- Community engagement and participation
			- Building trusting and communicative relationships with SCCY outside of the research
		- Inequity between adult researcher and vulnerable child	- Utilizing various recruitment approaches including flexible times to attend study site and access to services and care.
		- Equitable recruitment and chance to participate	
Respect for persons	Absence of a parent/guardian	- Who should provide informed consent for children/youth?	- Formal legal consent from governmental authorities
		- What type of consent is culturally and socially acceptable?	- Waiver of parental consent when appropriate
		- Do SCCY have the cognitive capacity to self-consent or provide assent?	- Informal community consent and approval
			- Approval from SCCY leaders
	Uncertain Cognitive Capacity	- How can cognitive capacity be assessed?	- Comprehension test to assess understanding of research and consent
		- Is the population literate? Can they read and write to provide consent/assent?	- Specially trained social worker
		- Is substance use a factor in cognitive capacity?	
		- SCCY are known for substance use, need to assess intoxication prior to participation.	
Beneficence	Coercion	- What type of compensation for participation is appropriate?	- Providing access to care and services regardless of participation
		- Lack of access to healthcare and basic necessities	- Adequate compensation for time away from the streets and/or transportation money
		- Power dynamics between research team and children	- Street outreach activities
	Child Protection	- How to protect children when social services and healthcare system infrastructure is weak?	- Establish protocol and procedure that work within the local social services and healthcare system
		- What types of care are available in the local setting?	- Have a dedicated social worker and psychologist on research team
		- Responsibilities to report abuse to authorities	- When feasible collaborate or form partnerships with local healthcare provider
		- Human rights violations and authorities as perpetrators	- Assess the local child protection system and report to authorities when in the best interest of the child

**Table 2:** Comprehension of Assent Test

Question	Evaluation of answers
*According to you, what is research?*	To test the basic and generalized knowledge the child has about the research study in terms of its being research as opposed to ‘pure’ clinical care. Words the research assistant is looking for include ‘knowledge’, ‘information’, ‘helping others’.
*What do you think will happen to you if you choose not to participate in the study?*	To see if the child understands that participation is voluntary and that she/he is free to withdraw at any time without consequence.
*If you choose to participate:*	1) To see if the child is able to mention any benefits he/she may experience from participating (e.g. seeing a doctor);2) To see if the child understands the risks she/he may encounter from participating (e.g. loss of confidentiality if they are in immediate danger to themselves or others, emotional distress)
*1) What are the good things that may happen to you?*	
*2) What are the bad things that may happen to you?*	
*Once you enroll in the study, how often will you have to participate?*	To test the child’s understanding of the frequency of visits during the study.
*What will happen to you during the research?*	To test the child’s understanding of procedures for the study, including the standardized interview and possible participation in a focus group.
*All the information you share with the research team will be confidential. What do you understand by this?*	To see if the child understands their relationship with the research team is based on trust, that personal information will be kept secret unless there are exceptional circumstances, specifically if the child is in immediate danger.

## Results

Ethical considerations, approaches & difficulties

Using the three research projects as a case study, we discuss the three categories of ethical considerations, the approaches we implemented, and difficulties we encountered to describe the processes of adapting ethical guidelines for SCCY’s specific vulnerabilities in LMIC.

### Justice: community participation & equitable recruitment

*Ethical Considerations:* Community engagement is essential for ethical research in LMIC [6, 32]; it respects the sociocultural context, and children’s lived experiences and perspectives [13]. Community participation supports the principle of justice and the balance of power between the researchers and community [6], as well as between the adult researcher and child participant [13]. Engaging SCCY in dialogue prior to commencing research upholds the principle of beneficence as it gives them a voice that may otherwise not be heard, and ensures that investigators avoid harm by taking into account their perspectives in relation to participation and procedures.

Equitable recruitment in research with hard to reach populations requires creative strategies to ensure that all possible members of a population who would benefit from the proposed research have the chance to participate. SCCY are a diverse population with varying levels of street-involvement and visibility. Children who spend only a portion of their time on the streets and street-involved girls may be especially difficult to identify and recruit due to their diminished visibility and extreme marginalization. They however represent important sub-populations that stand to benefit from targeted interventions.

*Approaches:* As part of our work, we involved all key stakeholders, from governmental officials, community-based organizations (CBOs), to community members and SCCY themselves.

At the outset of all three projects we involved the UG District (now County) Children’s Officer (DCO) to engage the community and to obtain approval to undertake the studies [22]. The DCO handles all children’s affairs in the county and is considered the *de facto* guardian of SCCY. Many SCCY do not have a legal guardian or have a guardian with limited involvement who cannot always be counted on to have the child's best interests at heart. Involvement of the DCO helped to ensure adequate protections of SCCY’s rights and welfare in the local setting.

We hosted a series of *mabaraza*, a traditional form of community assembly in Kenya used to disseminate information and make decisions, and in Kenya they are held as official public gatherings with Chiefs and sub-Chiefs [22, 33]. We conducted *mabaraza* with community members whom were residents living in these locations, SCCY, and street youth leaders to discuss the proposed studies and gather input on ethical considerations, and seek community approval. We used their input on factors involving the proposed research topics, informed consent processes, and types of compensation, which ensured we were safeguarding this vulnerable population and addressing their needs, while respecting their right to participation.

CBO’s provide drop-in and support services to SCCY and are locally run by community members. CBO’s working with SCCY identified priority areas of concern as substance use and sexual and reproductive health, which assisted in informing the research questions for Study 1 and Study 2. We conducted extensive outreach activities with SCCY to build relationships prior to commencing research. Through these interactions, we aimed to build trusting relationships with SCCY that respected their experiences and circumstances on the streets while engaging them in the research process.

We tried to maximize equitable recruitment through employing a street outreach worker and conducting outreach and recruitment at all known sites where SCCY congregate. We offered flexible times to attend the study clinic and Study 2 specifically provided foot care supplies for females at enrollment sites whether or not individuals chose to participate in the study. Girls were additionally offered a package of sanitary supplies, in order to address gender disparity in enrollment.

*Difficulties:* Some CBO’s expressed apprehension about conducting research with SCCY. To address this issue, we kept an open dialogue and communicated the value of research as an important component of informing their programs and services. We ensured study findings were translated back to CBO’s and policymakers working with SCCY through easily readable policy briefs and presentations.

During outreach activities SCCY consistently expressed need for assistance about many issues related to their health and well-being and reintegration into society. While we were unable to solve all of the issues, we took the time to listen and referred them to attend local service providers to seek available assistance. They also expressed that they felt no effective interventions have come from their participation in previous research. Establishing partnerships and collaborating with local organizations providing services to SCCY may assist in ensuring that effective interventions, as a result of research participation, are designed, implemented and responsive to SCCY’s needs.

Ensuring that equitable participation occurred among the sub-populations of SCCY was more problematic than expected. SCCY who work on the streets during the day and return home at night are hesitant to participate and take away time from their employment activities. While we identified a number of girls, many were unwilling or unable to participate. Solutions to these issues may be to have dedicated days at an enrolment site for sub-populations and to provide alternative options such as a mobile team to go to them in a suitable location.

### Respect for person: informed consent

*Ethical Considerations:* Children may not be developmentally competent to provide independent informed consent [10]. Their decision to participate requires a balance between respecting their autonomy and safeguarding their interests [13]. Children’s participation in the decision-making process is also informed by sociocultural values, and the informed consent and assent processes in LMIC require additional considerations [5, 7, 33].

For most types of research, guidelines recommend parental permission for a minor’s research participation [10, 13, 34]. In certain situations ethics committees can grant waivers to the requirement for parental permission. Specifically, adolescents are legally able to consent for certain types of treatments/interventions and minimal risk research (e.g. survey research) when the requirement for parental consent makes the research impracticable [35].

For SCCY it is generally not possible to safely obtain parental/guardian consent. Firstly, many of the children who live full-time on the streets have either no identified parent/guardian, or have no contact with parents/guardians [20, 31]. Children who are on the street during the day and return at night are frequently in neglectful and abusive situations, raising issues of safety. Secondly, for research on sensitive topics such as substance use and sexual reproductive health, parental permission may actually represent a risk, rather than a protection, and it introduces a risk of loss of confidentiality. Thirdly, while many SCCY may have adequate cognitive capacity to make autonomous decisions about their participation in research without a parent/guardian, in others, cognitive capacity is uncertain for a variety of reasons including: illiteracy, lack of formal education, substance use resulting in cognitive impairment, and very young age (<12 years).

*Approaches: Mabaraza* identified the community’s perception of street children’s capacity to provide informed consent and many felt street children could consent for themselves; however, community involvement and acceptance, the DCO providing legal consent, and the child’s age were important considerations [33]. Ensuring the community is informed and accepts that the research is going to be carried out was considered to be the first step in seeking informed consent for children to participate in research. Children older than 10 years were considered competent to make a decision regarding their participation in research.

**Fig. 1:**
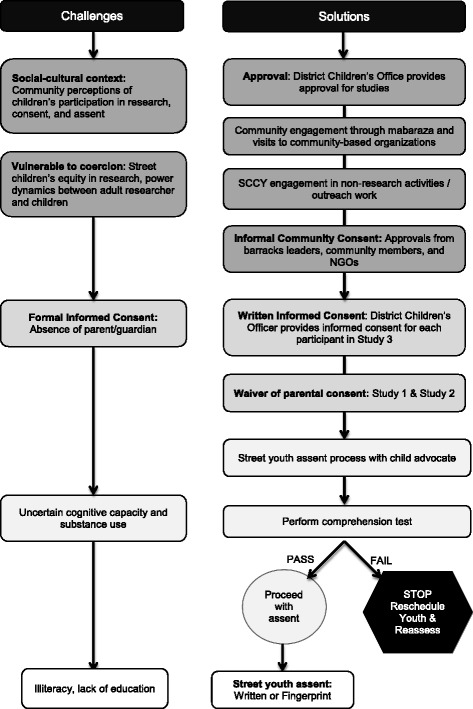
Challenges and solutions with informed consent process

Our overall process to gain informed consent and assent is presented in Fig. 1. Study 1 and 2 sought a waiver of individual guardian consent because as per human subjects regulations, the studies were minimal risk, the studies could not have been practicably carried out without the waiver, and because the waiver did not adversely alter the risk-benefit ratio for participants

Community-based approval for the studies was obtained from residents participating in *mabaraza*, CBO’s providing services to SCCY and the UG Children’s Forum. Through extensive outreach activities we sought general permission from street youth leaders to have SCCY who are members of their “bases/barracks” (specific gang that SCCY belong to) participate. In this situation it is informal community consent to gain entry and conduct research within this tight-knit community. The youth leaders provided their overarching informal consent to the researchers entering into their bases, to interact with the children and youth who are part of their group, and that they are agreeable and working with us in a participatory manner to ensure that the children are understanding the process of engaging in research and that we work together. The DCO provided legal consent to the studies occurring in UG County. In study 3, in lieu of parental/guardian permission, the DCO provided individual permission for each of the 100 SCCY to participate. This study had more extensive procedures and was longitudinal, and it was felt that the complexity warranted additional protection.

We designed an assent process to support SCCY to make an informed decision regarding their participation in the research that respected their autonomy and their right to dissent, while safeguarding them in situations where their capacity to make an informed decision was diminished. In all three studies, assent was obtained by a social worker trained in assenting vulnerable children. This social worker acted as a witness to the consent/assent process and documented it in a consent/assent note. If an individual was noted to be impaired as a result of substance use at the time of enrolment, they were asked to delay participation. Due to uncertain cognitive capacity, we assessed their ability to understand the nature of their participation in the research studies by undergoing a comprehension assessment (Table 2). SCCY were read the assent/consent form by the social worker, due to the high rates of illiteracy, in a language that was age and culturally appropriate. Participants who passed the comprehension test would proceed with written assent or consent (for those > 18 years in Study 2); those who failed had the option to be rescheduled and reassessed. Participants assented or consented by either signing their name or marking the paper with their fingerprint.

*Difficulties*: At times the DCO has minimal contact with SCCY in our setting, and the children and youth themselves don’t always view their office favorably. This office is governs child protection in the region and in theory, the DCO’s position is to protect vulnerable children in need within the county area he/she is assigned. However, in practice, sometimes children do fall through the cracks, particularly SCCY, due to an underdeveloped and resource-constrained child protection system. Selecting a suitable legal guardian to provide informed consent for SCCY’s participation and to safeguard their interests in LMIC, where their parents/guardians are not involved in their daily lives, and where parental permission may actually represent a risk, rather than a protection, remains a difficulty. A solution may be to allow SCCY to appoint a trusted adult community member to witness their assent for participation.

### Beneficence: vulnerability to coercion & responsibility to protect

*Ethical Considerations:* SCCY may be vulnerable to coercion due to the unequal power dynamic between them and the research team. As a population that lacks basic necessities and access to care, it is difficult to determine what if any type of incentive would sway participation. Some argue that even the smallest incentive for children and youth living deprived of any possessions is an inducement for participation [16, 18]. Compensation for SCCY’s participation in research may be material (money, food, clothing) or non-material (access to medical care and social services) [16], and should take into consideration the sociocultural context [13]. Monetary reimbursements may not be suitable due to the high levels of substance use [2], however we have found that SCCY’s first priority when money is available is food [31].

It is considered the duty of researchers to report suspected cases of child abuse and harm [36]. SCCY may disclose illegal activities, or that they are being harmed or abused during the research processes [9, 16]. In these cases researchers need to ensure an appropriate response that protects the child’s confidentiality and refers them to the appropriate services available [13]. In many LMIC such as Kenya, the authorities and child protection services are not adequately developed and equipped to deal with the complexities and abuses that SCCY face, and in some cases may be perpetrating human rights violations [37, 38], placing them at further risk when reporting.

*Approaches:* We used three approaches to minimize SCCY’s vulnerability to coercion. First, we employed street outreach workers and accompanied them to the streets to extensively interact with SCCY. This gave SCCY the opportunity to ask questions about the study and other issues in a comfortable environment with no pressure, while building the trust and rapport necessary for ethical research. This process occurred over several weeks prior to commencing research and was on-going throughout the duration of the studies. Second, we approached the issue of incentives for participation in terms of reimbursement [39]. Reimbursement for participation was considered as compensation for the time away SCCY took from their work on the streets to participate, and transportation to get to the study clinic, which upheld the principle of justice. We used non-monetary compensation in the form of milk and bread (Study 1 and 3), and a gift of 2 pens and a workbook (Study 2 and 3). Study 2 also monetarily compensated SCCY for transportation to the study site (50 KES, USD 0.70). Thirdly, SCCY requesting or requiring healthcare but who were not eligible to participate in the studies or who dissented were provided with healthcare services without enrolment. This addresses an important potential vulnerability of research participants in LMIC, the ability to access health care resources only through research participation [4]. These approaches to compensation as reimbursement for time and effort is consistent with best practices [39], and is a sign of respect to youth.

In situations where SCCY reported abuse or required psychosocial assistance, regardless of participation, an onsite psychologist assessed the individual and referred them to the best available standard of care. In our setting, that included being able to refer children and youth to the Centre for Assault and Recovery of Eldoret, run by MTRH in partnership with AMPATH. We established a protocol that included addressing each case on an individual basis, providing psychological support, reporting to authorities when appropriate, and ensuring that SCCY received any needed medical care while protecting their privacy.

*Difficulties:* Determining an appropriate form of compensation for research participation is challenging. It requires careful consideration of difficult the context and environment in which SCCY live as well as their substance use. In Study 3, items such as the pens and paper while well received, were not necessarily suitable as SCCY often have no place to store these items safely. Providing compensation such as tea and bread and monetary compensation for travel to the clinic site were deemed suitable and not coercive in our context, based on the amounts of money that street children earn on a daily basis in our setting [31].

Providing adequate protection and care for SCCY who report abuses and rights violations is difficult in higher income countries, and proved to be very difficult in LMIC. While the research protocol may plan to refer identified cases of harm and abuse to the appropriate authorities as required by institutional review boards, in reality this can be extremely challenging without an adequately developed child protection system.

## Discussion

Our interrelated studies provide examples of how to innovatively adapt existing ethical guidelines to safeguard SCCY in research while upholding their right to participate. However, ethically engaging SCCY in research in LMIC is not without significant challenges. Based on the successful approaches and difficulties presented as part of this case study, we have developed key recommendations for investigators conducting research with SCCY in LMIC to consider and adapt for their context.

We recognize that this article has strengths and limitations. Strengths include that all of the strategies that were implemented ensured the principles of beneficence, justice, and respect for persons were upheld during the research process with this vulnerable population. Secondly, our adaptation of ethical guidelines and innovative strategies to safeguard street-connected children and youth participating in research were successful in all three studies demonstrating their effectiveness across different types of research. However, a limitation of this article is external validity given that our approaches are situated within the socioeconomic and cultural context of Kenya. Yet, we believe that our ethical considerations and approaches provide an important starting point for any researcher to use as a framework for ethical research with street-connected children and youth within low- and middle-income settings. While our exact approaches may not the applicable in other countries, our key recommendations provide suggestions that can be adapted and applied in any setting.

### Key recommendations

First, and consistent with best practices for ethical research in LMIC [6], community engagement and working within the local sociocultural context were at the core of successfully conducting ethical research with SCCY in our setting. Investigators seeking to conduct research with SCCY in LMIC should assess the local context, to determine what organizations and services are available to SCCY, to establish relationships with organizations working with SCCY (when they exist), and determine what government child protection systems are in place and what their role in the research process may be. We recommend, when feasible, embedding research into existing systems to build local capacity to assist SCCY through implementation research. Embedding research within existing programs and services reduces coercion to participate to receive access to care or other incentives. Similarly, we propose that investigators consider providing non-research related support, especially in settings where no CBO’s exist to assist SCCY and social services and healthcare system infrastructure is weak.

Second, utilizing a multifaceted approached to informed consent and assent with SCCY was successful in safeguarding potential participants with diminished capacity while ensuring that children have the right to participate in matters affecting them. Gaining formal consent from the DCO for research activities to occur and informal community consent from CBO’s and SCCY leaders ensured that we respected the local sociocultural context. In our setting it was important to gain informal consent from stakeholders such as NGOs, and SCCY leaders who have increased interaction and involvement in SCCY’s lives than the DCO on a daily basis. This practice is consistent with the Society for Adolescent Health and Medicine’s guidelines for community consent when parental consent is not feasible or does not provide appropriate protections [14]. We recommend that researchers consider obtaining informal and formal consent from the community for the research activities to occur. It provides an additional protective mechanism and respects the local sociocultural context. Investigators should carefully determine who is the legal guardian of SCCY in their setting and determine who should act as a guardian to provide informed consent when feasible and appropriate. We suggest that children and youth should have the option of selecting a community member whom they trust to witness their informed consent if this is feasible and appropriate within the research setting. Finally, we advise researchers to make use of a comprehension test to provide an additional mechanism of safeguarding SCCY that may have diminished capacity to make an informed decision in the assent/consent process.

Third, in situations where children report abuses and rights violations, we recommend the investigators follow the core ethical principal of non-maleficence and assess each situation on a case-by-case basis. First, when appropriate, available, and when authorities did not perpetrate violations, investigators should follow the local procedures to report the case through the child protection system. Second, human rights violations should be documented for advocacy purposes and brought to the attention of international human rights organizations. Third, researchers should ensure that the participant receives the local standard of care. Fourth, we recommend that the research team include a social worker and psychologist when conducting research with this vulnerable population. Lastly, establishing partnerships with local hospitals and care providers, when feasible, can ensure that the participant can be referred for additional care, such as specialized facilities for sexual and gender-based violence.

Fourth, we recommend empowering SCCY by employing peer outreach workers to support research and non-related activities. Peer workers enable the community of children and youth in street circumstances to be active participants in the research. The use of peer workers provides additional ethical protection, as they can provide critical feedback about specific research procedures and approaches.

Lastly, we propose that the ERIC Guidance and the Society of Adolescent Health *Guidelines for Adolescent Health Research* consider including specific guidelines and approaches to ethically conducting research with children and youth in street circumstances in both developed and developing countries.

## Conclusions

The specific vulnerabilities of children and youth in street circumstances participating in research activities can be supported through innovative procedures. SCCY have complex physical and psychosocial health outcomes that require creative solutions that reflect their lived experience and the social, cultural, and economic context. The exclusion of SCCY from research, due in large part to the complexities in working with a population with multiple vulnerabilities, represents yet another injustice towards these marginalized and vulnerable youth.

Our studies demonstrate that it is possible to engage children and youth connected to the streets in LMIC in research while respecting their right to participate in issues affecting them, and upholding the core ethical principles of respect for persons, beneficence, and justice. By working within the local contexts, engaging relevant community members and leaders, and following best practices guidelines for vulnerable populations, international researchers can responsibly engage children and youth in street circumstances in ethical research with the goals of reducing inequities and advancing their health and human dignity.

## Abbreviations

LMIC: low- and middle-income countries
SCCY: street-connected children and youth
ERIC Project: Ethical Research Involving Children project
UG: Uasin Gishu
MU: Moi University
MTRH: Moi Teaching and Referral Hospital
AMPATH: Academic Model Providing Access to Healthcare
FGD: focus group discussions
IU: Indiana University
IRB: Institutional Review Board
IREC: Institutional Research Ethics Committee
CBOs: community-based organizations
DCO: District Children’s Officer
